# The Percentage of Militarily Inefficients

**Published:** 1919-01-25

**Authors:** 


### THE PERCENTAGE OF MILITARILY INEFFICIENTS.

THE necessity of examining vast numbers of the men of these islands to determine the fitness of our manhood for war has revealed a percentage of unfit men so enormous as to startle the community from the apathy with which it had regarded such matters. There is no reason to suppose that a similar examination of our women would not show us as great a proportion of physically and mentally defective persons. The cause of the inefficiency is variously stated; too much work, too much drink, too much venereal disease, too little exercise, too little fresh air, too small wages, too little food, too much food, bad houses—it depends on the idea most prominent in the mind of the writer.

These causes all may produce some of the inefficiency, but probably the main cause is the one cause that is usually carefully left out of count. That cause is the breeding from hereditarily weak and feeble and defective men and women, and carefully keeping alive their offspring. The British Isles have a magnificent population if the better half were all the population. The British Isles would probably have the rottenest population in the world if they had only the worst half of the population. That bad half is carried on the backs of the better half, an old man of the mountain choking, strangling, and draining it. No farm, no stud, no kennel could keep up its average of stock if the worst and weediest were encouraged to breed equally as freely as the best. No matter how much it may jar, human beings are animals and subjected to hereditary influences as much as animals. Black cats have black kittens, and feeble, inefficient people have feeble, inefficient sons and daughters. The occasional exception does but emphasise the rule—the solution of the problem may be in the lap of the gods or in the lap of a Ministry of Health, but neither the gods nor a Ministry can breed strong stock from feeble stock without the severest selection.

